# Epidermal growth factor receptor (EGFR) is an independent adverse prognostic factor in esophageal adenocarcinoma patients treated with cisplatin-based neoadjuvant chemotherapy

**DOI:** 10.18632/oncotarget.2268

**Published:** 2014-07-26

**Authors:** Michaela Aichler, Martin Motschmann, Uta Jütting, Birgit Luber, Karen Becker, Katja Ott, Florian Lordick, Rupert Langer, Marcus Feith, Jörg Rüdiger Siewert, Axel Walch

**Affiliations:** ^1^ Research Unit Analytical Pathology- Institute of Pathology, Helmholtz Zentrum München, German Research Center for Environmental Health, Ingolstaedter Landstraße 1, Neuherberg, Germany; ^2^ Institute of Computational Biology, Helmholtz Zentrum München, German Research Center for Environmental Health, Ingolstaedter Landstraße 1, Neuherberg, Germany; ^3^ Institute of Pathology, Technische Universität München, Trogerstraße 18, München, Germany; ^4^ Department of Surgery, University Hospital of Heidelberg, Im Neuenheimer Feld 110, Heidelberg, Germany; ^5^ University Cancer Center Leipzig, University Clinic Leipzig, Liebigstraße 20, Leipzig, Germany; ^6^ Department of Surgery, Klinikum rechts der Isar, Technische Universität München, Ismaninger Straße 22, München, Germany; ^7^ Directorate, University of Freiburg, Hugstetter Straße 55, 79106 Freiburg, Germany

**Keywords:** EGFR, prognosis, chemotherapy, cisplatin, esophageal cancer

## Abstract

Neoadjuvant platin-based therapy is accepted as a standard therapy for advanced esophageal adenocarcinoma (EAC). Patients who respond have a better survival prognosis, but still a significant number of responder patients die from tumor recurrence. Molecular markers for prognosis in neoadjuvantly treated EAC patients have not been identified yet. We investigated the epidermal growth factor receptor (EGFR) in prognosis and chemotherapy resistance in these patients. Two EAC patient cohorts, either treated by neoadjuvant cisplatin-based chemotherapy followed by surgery (n=86) or by surgical resection (n=46) were analyzed for EGFR protein expression and gene copy number. Data were correlated with clinical and histopathological response, disease-free and overall survival.

In case of EGFR overexpression, the prognosis for neoadjuvant chemotherapy responders was poor as in non-responders. Responders had a significantly better disease-free survival than non-responders only if EGFR expression level (p=0.0152) or copy number (p=0.0050) was low. Comparing neoadjuvantly treated patients and primary resection patients, tumors of non-responder patients more frequently exhibited EGFR overexpression, providing evidence that EGFR is a factor for indicating chemotherapy resistance.

EGFR overexpression and gene copy number are independent adverse prognostic factors for neoadjuvant chemotherapy-treated EAC patients, particularly for responders. Furthermore, EGFR overexpression is involved in resistance to cisplatin-based neoadjuvant chemotherapy.

## INTRODUCTION

Esophageal adenocarcinoma (EAC) is among the most rapidly increasing malignancies in the western world [1-3]. Despite improvement in surgical outcomes, the prognosis for EAC remains dismal with less than 20% of patients surviving 5 years [2]. Neoadjuvant chemo- or radiochemotherapy followed by resection has been shown to provide a survival benefit for patients with locally advanced EAC compared to surgery alone [4-7]. A common therapeutic approach for these EAC is a multimodal treatment that includes preoperative application of cis-diamminedichloroplatinum II (cisplatin) and 5-flurouracil (5-FU) chemotherapy, followed by resection [4, 6-9]. Patients who respond to the neoadjuvant chemotherapy have a better survival [9-11]. Response evaluation for EAC treatment can be performed according to metabolic response evaluation using fluordeoxyglucose positron emission tomography, clinical response evaluation by endoscopy, endoluminal ultrasound and computed tomography (CT) scans, or histopathological response evaluation following resection. Until now, the histological response evaluation has been considered the gold standard [12, 13], and these results are strongly associated with survival [14-16]. It is generally accepted that patients with a primary tumor response have a significantly improved prognosis compared to patients who do not respond [17]; however, despite a positive histological response, a significant percentage of patients still die due to tumor recurrence [16, 18, 19]. The reasons for this remains unclear, and effective tools to predict the clinical course of patients treated with neoadjuvant chemotherapy are missing. The Union for International Cancer Control/American Joint Committee on Cancer (UICC/AJCC) TNM classification (7^th^ edition) provides a good prognostic stratification for primary resected esophageal cancer, but the accuracy of this current classification in neoadjuvant chemotherapy-treated EAC remains largely unknown [13]. Therefore molecular markers that may determine the prognosis in neoadjuvant chemotherapy-treated EAC patients are urgently needed.

The epidermal growth factor receptor (EGFR) pathway has been recognized as one of the key proliferative pathways that is dysregulated during tumorigenesis, and this pathway is a major regulator of various signaling pathways involved in cell survival, migration, and tissue regeneration [20]. In adenocarcinomas of the esophago-gastric junction (AEG) and the distal esophagus, EGFR expression has been found in approximately 30–60% [21, 22], while *EGFR* gene amplification has been found in 8–31% [23, 24]. Although an association of EGFR expression or *EGFR* gene amplification with poor prognosis in primary resection patients has been proposed [21, 25], the clinical and biological significance of such expression in neoadjuvantly treated patients remain undefined. To further elucidate the role of EGFR in EAC, we examined the EGFR protein expression and gene copy number changes, clinical characteristics, and outcome in EAC patients treated with cisplatin-based neoadjuvant chemotherapy. We hypothesized that EGFR in EAC promotes an aggressive tumor phenotype and results in poor outcomes and neoadjuvant chemotherapy resistance.

## RESULTS

### Correlation of EGFR protein expression, copy number changes, and clinical variables

Tissue samples of 86 patients who underwent neoadjuvant chemotherapy and 46 samples of patients treated by primary resection without chemotherapy were examined for EGFR protein expression and gene copy number changes (*EGFR*-to-chromosome-7 ratio) (Table 1). EGFR expression and copy number changes were significantly positively correlated in neoadjuvant chemotherapy-treated patients (p<0.0001) and primary resection patients (p=0.0057). High EGFR protein expression (IHC score 2+/3+) occurred in 31.4% of patients who received neoadjuvant chemotherapy. In contrast, only 13.0% of patients in the primary resection group demonstrated high EGFR protein expression (Table 1). The number of patients with an *EGFR* gene copy number amplification (*EGFR*-to-chromosome-7 ratio of >2.2) in neoadjuvant chemotherapy-treated patients (4.7%) was comparable to that in the primary resection patient group (4.3%; Table 1).

**Table 1:** Characteristics of the neoadjuvant chemotherapy-treated and primary resection cohorts

	EGFR			EGFR/Chromosome-7 ratio		
	IHC Score					
	0/1+	2+/3+	total	≤2.2	>2.2	total
Neoadjuvant chemotherapy-treated EAC (cT3/cT4)						
**Number of patients**						
Total	59	27	86	82	4	86
	(68.6%)	(31.4%)		(95.3%)	(4.7%)	
**Tumor stage**						
ypT1	5	0	5	5	0	5
	(100.0%)	(0.0%)		(100.0%)	(0.0%)	
ypT2	15	6	21	19	1	20
	(71.4%)	(28.6%)		(95.0%)	(5.0%)	
ypT3	30	17	47	45	2	47
	(63.8%)	(36.2%)		(95.7%)	(4.3%)	
**Lymph node metastasis**						
ypN0	17	4	21	21	0	21
	(81.0%)	(19.0%)		(100.0%)	(0.0%)	
ypN1	33	19	52	49	3	52
	(63.5%9	(36.5%)		(94.2%)	(5.8%)	
**Distant metastasis**						
M0	45	21	66	63	3	66
	(68.1%)	(31.9%)		(95.6%)	(4.4%)	
M1	5	2	7	7	0	7
	(71.4%)	(28,6%)		(100.0%)	(0.0%)	
**Resection**						
R0	40	18	58	56	3	59
	(69.0%)	(31.0%)		(94.9%)	(5.1%)	
R1	10	3	13	12	0	12
	(76.9%)	(23.1%)		(100.0%)	(0.0%)	
R2	0	2	2	2	0	2
	(0.0%)	(100.0%)		(100.0%)	(0.0%)	
**ypUICC**						
1	3	0	3	3	0	3
	(100.0%)	(0.0%)		(100.0%)	(0.0%)	
2	13	4	17	16	0	16
	(76.5%)	(23.5%)		(100.0%)	(0.0%)	
3	8	3	11	10	1	11
	(72.7%)	(27.3%)		(90.9%)	(9.1%)	
4	21	14	35	33	2	35
	(60.0%)	(40.0%)		(94.3%)	(5.7%)	
5	5	2	7	7	0	7
	(71.4%)	(28.6%)		(100.0%)	(0.0%)	
**Responder**	15	4	19	18	1	19
	(78.9%)	(21.1%)		(94.7%)	(5.3%)	
**Non-responder**	33	17	50	49	1	50
	(66.0%)	(34.0%)		(98.0%)	(2.0%)	
**Primary resection EAC (cT3)**						
**Number of patients**						
Total	40	6	46	44	2	46
	(87.0%)	(13.0%)		(95.7%)	(4.3%)	
**Tumor stage**						
pT3 (total)	40	6	46	44	2	46
	(87.0%)	(13.0%)		(95.7%)	(4.3%)	
**Lymph node metastasis**						
pN0	10	0	10	10	0	10
	(100.0%)	0%		(100.0%)	(0.0%)	
pN1	30	6	36	34	2	36
	(83.3)	(16.7%)		(94.4%)	(5.6%)	
**Distant metastasis**						
M0	36	4	40	39	1	40
	(90.0%)	(10.0%)		(97.5%)	2.5%)	
M1	4	2	6	5	1	6
	(66.7%)	(33.3%)		(83.3%)	(16.7)	
**Resection**						
R0	26	1	27	27	0	27
	(96.3%)	(3.7%)		(100.0%)	(0.0%)	
R1	13	4	17	16	1	17
	(76.5%)	(23.5%)		(94.1%)	(5.9%)	
R2	0	1	1	1	0	1
	(0.0%)	(100.0%)		(100.0%)	(0.0%)	

### EGFR overexpression is an independent adverse prognostic factor in EAC patients

**Figure 1:**
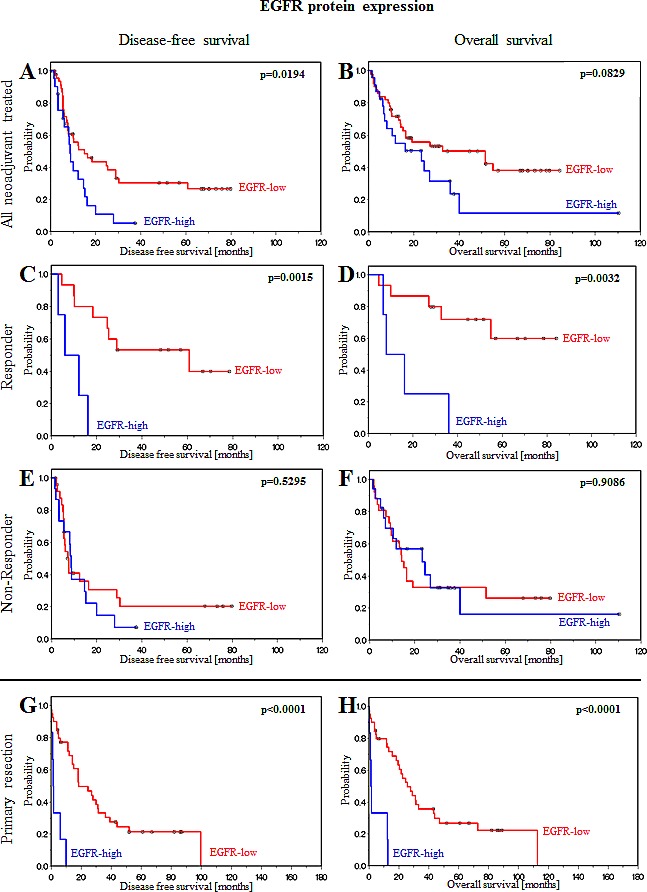
EGFR protein expression is associated with prognosis in patients treated with neoadjuvant chemotherapy or primary resection (A) Disease-free and (B) overall survival of all neoadjuvant chemotherapy-treated patients. (C) Disease-free and (D) overall survival of responding patients. (E) Disease-free and (F) overall survival of non-responding patients. (G) Disease-free and (H) overall survival of primary resection patients. Patients can be stratified as patients with a good survival prognosis if EGFR protein expression is low and patients with a poor survival prognosis if EGFR protein expression is high.

EGFR protein expression was significantly associated with outcome in the whole population including neoadjuvant treated and primary resected patients (Figure 1, Table 2). In the neoadjuvant treated group, EGFR protein expression was significantly associated with disease-free survival (p=0.0194; Figure 1A, Table 2). In primary resection patients, a significant association between EGFR protein expression and disease-free survival (p<0.0001; Figure 1G, Table 2) and overall survival (p<0.0001; Figure 1H, Table 2) was identified. Further stratification of the neoadjuvant chemotherapy group into responding and non-responding patients revealed that survival of patients who did not respond to chemotherapy was not associated with EGFR expression (Figure 1E-F, Table 2). In contrast, disease-free and overall survival of responding patients were significantly associated with the EGFR expression status (Figure 1C-D, Table 2). Responding patients with low EGFR expression profit significantly in terms of disease-free (p=0.0015; Figure 1C, Table 2) and overall survival (p=0.0032; Figure 1D, Table 2), while high EGFR expression significantly shortened survival in this subgroup (Figure 1C-D, Table 2).

**Table 2:** Correlation of EGFR protein expression and copy number changes with patient survival data

	EGFR		EGFR/Cep7	
	protein expression		Ratio	
	EGFR-low vs EGFR-high		≤2.2 vs >2.2	
	P	n =	P	n =
Disease-free survival				
Neoadjuvant chemotherapy-treated EAC (cT3/cT4)	0.0194	71	0.7531	71
Responder	0.0015	19	0.0359	19
Non-responder	0.5295	43	0.6685	43
Primary resection EAC (cT3)	<0.0001	46	0.8006	46
Overall survival				
Neoadjuvant chemotherapy-treated EAC (cT3/cT4)	0.0829	73	0.3820	73
Responder	0.0032	19	0.0359	19
Non-responder	0.9086	43	0.4451	43
Primary resection EAC (cT3)	<0.0001	46	0.7494	46

Multivariate Cox regression analysis confirmed EGFR overexpression as an independent adverse prognostic factor for disease-free survival (p=0.0050; HR=24.004; 95% confidence intervals (CI), 2.612–220.622) and overall survival (p=0.0033; HR=13.466; 95% CI, 2.383–76.078) in patients who responded to neoadjuvant chemotherapy (Table 3). This effect was also observed in primary resection EAC patients with respect to disease-free (p=0.0296; HR=1.994; 95% CI, 1.071–3.716) and overall survival (p=0.0448; HR=1.899; 95% CI, 1.015–3.555; Table 3).

**Table 3:** Stepwise Cox regression analysis and hazard ratios of disease-free and overall survival with prognostic factors in neoadjuvant chemotherapy-treated and primary resection EAC patients

	Univariate	Multivariate	HR	95% Confidence Intervals
Disease-free survival				
Neoadjuvant chemotherapy-treated EAC				
UICC staging	0.0001	0.0002	1.848	1.339 – 2.551
Responder				
EGFR protein expression	0.0082	0.0050	24.004	2.612 – 220.622
ypM	<0.0001	0.0036	58.135	3.785 – 892.811
Non-responder				
ypM	0.0236	0.0309	2.942	1.104 – 7.839
Primary resection EAC				
R	0.0459	0.0208	2.414	1.144 – 5.095
ypN	0.0300	0.0140	3.329	1.275 – 8.690
EGFR protein expression	0.0258	0.0296	1.994	1.071 – 3.716
Overall survival				
Neoadjuvant chemotherapy-treated EAC				
ypM	<0.0001	0.0243	3.557	1.179 – 10.734
EGFR protein expression	0.0491	0.0483	1.962	1.005 – 3.829
UICC staging	0.0451	0.0488	1.512	1.002 – 2.282
Responder				
ypM	0.0012	0.0017	45.854	4.206 – 499.865
EGFR protein expression	0.0037	0.0033	13.466	2.383 – 76.078
Non-responder				
UICC staging	0.0095	0.0016	2.303	1.371 – 3.869
Primary resection EAC				
R	0.0131	0.0047	3.111	1.415 – 6.838
ypN	0.0205	0.0137	3.578	1.298 – 9.862
EGFR protein expression	0.0405	0.0448	1.899	1.015 – 3.555

Abbreviation: HR, hazard ratio

Thus, EGFR protein expression is an independent adverse prognostic factor, in particular, patients who respond to neoadjuvant chemotherapy can be stratified into patients with a good survival prognosis with low EGFR protein expression and patients with a poor survival prognosis with high EGFR protein expression.

**Figure 2:**
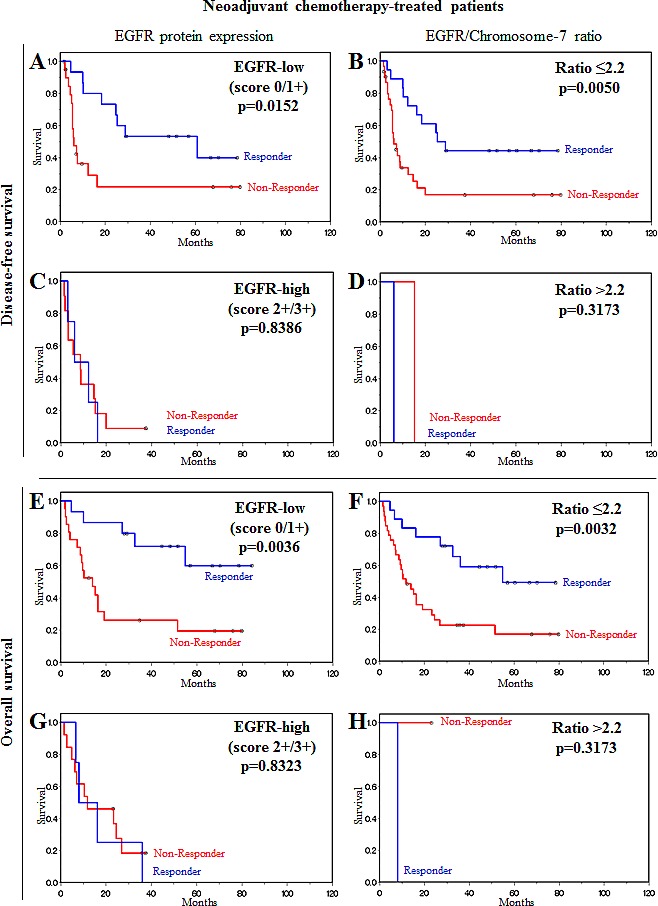
EGFR overexpression and *EGFR*/Chromosome-7 ratio are molecular factors for stratification of patients after neoadjuvant chemotherapy (A, B, C, D) Disease-free survival of neoadjuvant chemotherapy-treated patients. (A) Low EGFR protein expression. (B) Low *EGFR*/Chromosome-7 ratio. (C) High EGFR protein expression. (D) High *EGFR*/Chromosome-7 ratio. (E, F, G, H) Overall survival of neoadjuvant chemotherapy-treated patients. (E) Low EGFR protein expression. (F) Low *EGFR*/Chromosome-7 ratio. (G) High EGFR protein expression. (H) High *EGFR*/Chromosome-7 ratio. Prognosis for neoadjuvant chemotherapy responders was as poor as that for non-responders when EGFR expression level was high. Responders had a significantly better prognosis than non-responders when EGFR expression level or *EGFR*/Chromosome-7 ratio were low.

We further evaluated the influence of EGFR protein expression in neoadjuvant chemotherapy-treated patients as a molecular factor for survival prognosis. A statistically significant association between low EGFR expression levels (EGFR-low) and disease-free survival (p=0.0152) and overall survival (p=0.0036) was noted in responders and non-responders (Figure 2A), although responders had a significantly better prognosis than non-responders. In cases where patients did not respond to chemotherapy but had low EGFR protein expression, after surviving the first two years after therapy, their disease-free and overall survival was as good as that for responder patients. In contrast to these findings, in patients with high EGFR expression, the prognosis for responders was as poor as that for non-responders (Figure 2C). Thus, neoadjuvant therapy responders with a putative good prognosis were identified as actually having a poor prognosis after neoadjuvant therapy according to EGFR overexpression.

We also evaluated these findings with regard to copy number changes (*EGFR*-to-chromosome-7 ratio) and obtained similar results. A statistically significant difference was observed in responders and non-responders between disease-free survival (p=0.0050) and overall survival (p=0.0032) and patients who did not have an *EGFR* amplification (Figure 2B), and a significantly better prognosis was predicted for responders than for non-responders. Identification of responder patients with a poor prognosis as for EGFR-high patients was not possible using FISH analysis in patients with an *EGFR*-to-chromosome-7 ratio >2.2 due to limitations in patient numbers (Figure 2D).

### Comparison of clinical, histopathological response, and EGFR expression as factors for prognosis

We examined EGFR protein expression with respect to clinical and histopathological response evaluation as a factor for prognosis in the neoadjuvant chemotherapy-treated patient group. Multivariate COX regression analysis revealed that EGFR protein expression was the strongest prognostic factor for disease-free survival (p=0.0218; HR=2.010; 95% CI, 1.107–3.650), while histopathological response was the strongest factor for overall survival (p=0.0018; HR=3.626; 95% CI, 1.613–8.154).

### Comparison of EGFR overexpression in neoadjuvant chemotherapy and primary resection EAC patients

**Figure 3:**
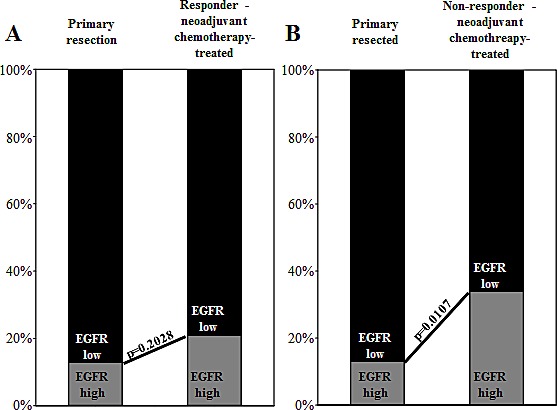
**Bar graphs depict EGFR expression level distribution (EGFR-high vs.** EGFR-low) in comparisons of primary resection patients with neoadjuvant chemotherapy (A) responders and (B) non-responders. EGFR overexpression is more frequent in non-responding patients, and thus, this overexpression can be interpreted as a factor for chemotherapy resistance.

The frequency of EGFR overexpression in patients who respond to neoadjuvant chemotherapy was compared with the frequency of EGFR overexpression in EAC patients treated with primary resection without chemotherapy. The percentage of cases with high EGFR expression levels (score 2+ or 3+) was significantly more often found (p=0.0108) in the neoadjuvant chemotherapy-treated cohort (31.4%, 27 of 86 vs. 13.0%, 6 of 46) compared to the primary resection cohort (Table 1). To further assess these findings, we separately compared the responder and the non-responder subgroup with the primary resection cohort. No significant difference in the frequency of EGFR overexpression was detected between chemotherapy-responders and in primary resection patients (p=0.2028; Figure 3A). In contrast, the frequency of EGFR overexpression, was significantly higher in non-responders than in primary resection patients (p=0.0107; Figure 3B).

## DISCUSSION

Neoadjuvant platinum-based chemotherapy is a standard treatment regimen for patients with advanced EAC [4, 7]. It is generally accepted that patients who respond to neoadjuvant chemotherapy have a survival benefit [10, 11]; however, despite a positive histological response, a significant number of patients die from tumor recurrence [16, 18, 19]. Therapeutic response of patients often do not prolong patient survival [26]. This might be due to current therapies eliminate abundant cancer cells but do not target stem cells [26]. In a review of concept, Blagosklonny pointed out further explanations [27]. The response-survival paradox could be explained as effective therapy selects for resistance among proliferating cancer cells [27]. Mechanisms of resistance might be either based nononcogenic (e.g. drug transporters and mutation in drug-targets) or oncogenic (e.g. apoptosis and cell cycle dysregulation) [27]. The oncogenic resistance is associated with highly aggressive cancer phenotype and, therefore no survival benefit [27]. The above mentioned theories might give an explanation for tumor recurrence in responding EAC patients. In the current study, we investigated EGFR in prognosis and chemotherapy resistance esophageal adenocarcinoma patients with a positive response to therapy but who died from tumor recurrence. EGFR, a receptor tyrosine kinases, is a major regulator of signaling pathways involved in cell survival, migration, and tissue regeneration [20]. Several studies have demonstrated that EGFR expression is a strong prognostic indicator that correlates with both higher recurrence rates and shorter survival in different tumor entities [21, 25, 28-30]. Additionally, studies on the EGFR status as a prognostic factor in patients treated by neoadjuvant chemotherapy have been reported [31-33]. These studies have yielded contradictory results across different cancer types [31-33]. In a recently published study, EGFR overexpression was useful in predicting response in patients with triple-negative breast cancer treated with neoadjuvant chemotherapy [33]. In an earlier study of triple-negative breast cancer, patients with EGFR-positive had a less favorable prognosis and a poorer response to neoadjuvant chemotherapy than patients with EGFR-negative tumors [32]. On the other hand, in patients with locally advanced rectal cancer treated with preoperative chemoradiotherapy, *EGFR* gene copy number was neither predictive nor prognostic [31]. A few studies have investigated EGFR expression in EAC patients, but these studies have focused on prognosis in patients with primary resection of EAC or AEG. Although in these studies EGFR was associated with poor overall survival, a consistent association between EGFR and an adverse outcome was not yet substantiated [21, 25, 29, 30, 34]. The new finding in our study is, that EGFR expression predicted poorer survival outcomes in a subgroup of EAC patients with initial histopathologic response to chemotherapy. To the best of our knowledge, this was not yet studied before in any other population. In addition, in our study population of primary resection patients, EGFR expression was a strong and independent prognostic factor for disease-free and overall survival. A recently published study investigated factors that predict prognosis and recurrence in patients with esophagogastric adenocarcinoma and a histopathological response with less than 10% residual tumor [18]. Response of the primary tumor did not ensure recurrence-free long-term survival, although complete histopathological responders had a better prognosis compared to partial responders [18]. Another recently published study defined a multifactorial histopathological score based on ypT category, ypN, and degree of histological tumor regression for the prediction of prognosis of resected EAC after cisplatin-based neoadjuvant chemotherapy [35]. Our data, however, demonstrated that EGFR expression or copy number changes are strong and independent molecular prognostic factors for disease-free and overall survival in patients with EAC who responded to neoadjuvant chemotherapy but had an unfavorable diagnosis.

Cisplatin is a DNA-damaging anti-tumor agent that activates nuclear as well as cytoplasmic signaling pathways involved in regulation of the cell cycle, damage repair, and programmed cell death [36]. EGFR expression may be involved in resistance to cisplatin [37, 38]. The signals generated by DNA damage caused by cisplatin treatment modulate EGFR activity in EGFR-expressing cells and suppress cell death by upregulating antiapoptotic proteins [37]. In addition, inhibition of EGFR activation enhances cisplatin-induced cell death [37]. Overexpression of EGFR and inhibition of proliferation has been observed in cisplatin-treated ovarian carcinoma cells, and these molecular changes were hypothesized to be an escape mechanism of tumor cells [39]. Another study demonstrated that treatment of chemosensitive neuroblastoma cells with cisplatin reversibly increased EGFR expression and that cisplatin-resistant cells exhibited enhanced EGFR expression dependent of the presence of cisplatin [40]. In squamous cell carcinoma cells, increased EGF signaling and subsequent increased interleukin (IL)-1ß contributed to chemotherapeutic resistance [41]. Furthermore, acquired cisplatin resistance in EGFR-expressing lung cancer cells did not affect the sensitivity to EGFR tyrosine kinase inhibitors [42]. Together, these studies demonstrate that EGFR may be involved in mechanism of resistance to cisplatin. In the present study, we compared the frequency of EGFR overexpression in primary resection patients to neoadjuvant chemotherapy responders and non-responders. In the non-responding subgroup, EGFR expression was significantly higher than that in chemotherapy responders as well as in the primary resection patients. These findings provide evidence that EGFR overexpression is a factor for chemotherapy resistance and supports the hypothesis that EGFR is involved in the mechanism of resistance against cisplatin-based neoadjuvant chemotherapy.

Resistance can be exploited for therapeutic advantage [43, 44]. Evolving from several decades of systematic research in cancer cell biology, several EGFR inhibitors, such as monoclonal antibodies and tyrosine kinase inhibitors have been developed and implemented in clinical application [45]. The efficacy of cetuximab in combination with chemotherapy was evaluated as a first-line treatment of advanced gastric and gastro-esophageal junction cancer [46-49]. The overall results of these studies revealed response rates between 41 and 65%. The efficacy of cetuximab plus capecitabin and cisplatin was subsequently tested in a multinational randomized phase III trial for the first-line treatment of patients with advanced gastric and gastro-esophageal junction cancer (EXPAND, NCT00678535) [50]. The addition of cetuximab to capecitabine-cisplatin provided no additional benefit to chemotherapy alone in the first-line treatment of advanced gastric or gastro-esophageal junction cancer; however, this study did not specifically focus on AEG 1 patients. Therefore, studies on the effect of EGFR-directed therapy in AEG 1 patients have been missing but may be of interest in the neoadjuvant chemotherapy setting especially for EGFR-positive patients.

In conclusion, our data demonstrate that EGFR overexpression and gene copy number changes are independent adverse prognostic factors in EAC patients and may be useful molecular markers for outcome prediction in patients who receive neoadjuvant platin-based chemotherapy.

## PATIENTS, MATERIALS AND METHODS

### Study population

In this study, a total of 132 patients with EAC (all classified as type I according to Siewert [51]) were included. The patients were staged as cT3 or cT4 based on endoscopic ultrasonography and CT of the chest and abdomen. The study population consisted of two different treatment groups: one was treated with cisplatin-based neoadjuvant chemotherapy and the second was treated by primary resection without neoadjuvant chemotherapy. All patients provided written informed consent, and patient data were acquired with approval from the ethics committee of the Technische Universität München, Germany. For all patients, surgical resection material (formalin-fixed paraffin-embedded tissue) was available.

#### Neoadjuvant chemotherapy cohort

Patients (n=86) were treated with neoadjuvant chemotherapy consisting of two cycles of cisplatin on days 1, 15, and 29 and folinic acid plus fluorouracil on days 1, 8, 15, 22, 29, and 36, all repeated on day 49, as previously described in detail [52].

#### Primary resection cohort

Patients (n=46) underwent primary abdominothoracic esophagectomy [53] without chemotherapy or radiotherapy. For comparison with the neoadjuvant chemotherapy-treated patient group, only patients staged as cT3 were included in the study.

### Response evaluation and follow-up

Tumor response to neoadjuvant chemotherapy was characterized clinically and histopathologically.

*Clinical response* was evaluated by endoscopy with endoluminal ultrasound and CT scan after the first and second cycle as described previously [54]. Response was defined as at least 50% reduction in the size of the primary tumor, as measured by endoscopy and imaging studies [55].

*Histopathological tumor regression* was assessed according to a recently published scoring system [56]. For the purposes of this study, all patients with fewer than 10% residual tumor cells (regression score 1) were classified as responders. All other patients were classified as histopathological non-responders.

*Patient follow-up* included endoscopy and CT of the chest and abdomen at 3-month intervals during the first year after surgery and thereafter at 6-month intervals. Overall survival and disease-free survival were calculated from the day of surgery. Survival analyses for the primary resection cohort were calculated from all 46 patients with a median follow-up of 19.9 months (range, 0.1 to 134.0 months) for overall survival and 14.5 months (range, 0.1 to 134.0 months) for disease-free survival. For the neoadjuvant chemotherapy-treated cohort, median follow-up was 17.0 months for overall survival (range, 0.8 to 110.4 months) and 9.2 months for disease-free survival (range, 0.1 to 79.8 months). The observation time was the interval between diagnosis and last contact (death or last follow-up).

### Tissue microarrays

Three representative areas of the tumor (1.0 mm diameter) were removed from paraffin-embedded tissue blocks, which had been prepared at the time of resection, using a tissue microarray instrument (Beecher Instruments, Sun Prairie, Wisconsin, USA). Serial sections were cut for the purpose of immunohistochemistry (IHC) and transferred to adhesive slides using the “paraffin-tape-transfer-system” according to manufacturer's instructions (Instrumedics, Hackensack, NJ, USA).

### Fluorescence *in situ* hybridization (FISH), evaluation, and image analysis

A commercially available assay with fluorescence-labeled locus-specific DNA probes for EGFR and chromosome-7 centromeric α-satellite (Chrombios) was hybridized with the samples according to a previously described protocol [57]. The hybridization specificity of the probes was tested with metaphase spreads, and lymphocytes served as internal controls in tumor tissue samples. Signal evaluation was performed by visual counting using an epifluorescence microscope equipped with a C-Apochromat 63x/NA 1.2 W objective and an AxioCam b/w charge-coupled device camera (Zeiss Axioplan 2, Carl Zeiss Microimaging GmbH; AxioVision software release 4.5) according to standard procedures. At least 50 tumor cells per case were selected randomly, whereas only cells with a minimum of one signal for *EGFR* gene and centromere-7 were chosen, and the mean was calculated. FISH scoring for *EGFR*-to-chromosome-7 ratio was applied, and two categories were determined: mean ratios of ≤2.2 and >2.2. Ratios ≤2.2 were designated as non-amplified, while ratios >2.2 were designated as *EGFR* amplification.

### Immunohistochemical analysis and reactivity score

EGFR immunohistochemistry was performed using the Dako EGFRpharmDx^TM^ assay detection system (Dako Diagnostika GmbH, Hamburg, Germany) as described previously [58]. Tumors were considered to be negative when no staining or membrane staining in <10% neoplastic cells was observed. Weak complete and/or incomplete membrane staining in >10% neoplastic cells was considered as 1+ positive, while moderate complete and/or incomplete membrane staining in >10% neoplastic cells was considered as 2+ positive. Strong complete and/or incomplete membrane staining in >10% neoplastic cells was considered as 3+ positive. Cases with a score of 0 or 1+ were considered as EGFR-low expression cases, while those with a score of 2+ or 3+ were considered overexpression (EGFR-high) cases.

### Statistical Analysis

Correlations between variables were calculated using the Pearson correlation coefficient. Frequency tables were tested by χ^2^ test or Fisher exact test for comparison of discrete variables. Stepwise Cox regression analysis was applied to select parameters for predicting the overall survival time or disease-free survival time, their calculated hazard ratio are listed. Kaplan-Meier survival curves for different strata were plotted for overall survival and disease-free survival. The differences were tested by the log-rank test. All statistical tests were performed with SAS statistical package version 9.2 (SAS Institute Inc., Cary, NC), and significance was determined at the 95% level.
